# Using automated text classification to explore uncertainty in NICE appraisals for drugs for rare diseases

**DOI:** 10.1017/S0266462323002805

**Published:** 2024-01-05

**Authors:** Lea Wiedmann, Jack Blumenau, Orlagh Carroll, John Cairns

**Affiliations:** 1 Department of Health Services Research and Policy, Faculty of Public Health and Policy, London School of Hygiene & Tropical Medicine, UK; 2 Department of Political Science, Faculty of Social & Historical Sciences, University College London, UK

**Keywords:** health technology assessment, supervised learning, uncertainty, rare diseases, National Institute for Health and Care Excellence

## Abstract

**Objective:**

This study examined the application, feasibility, and validity of supervised learning models for text classification in appraisals for rare disease treatments (RDTs) in relation to uncertainty, and analyzed differences between appraisals based on the classification results.

**Methods:**

We analyzed appraisals for RDTs (*n* = 94) published by the National Institute for Health and Care Excellence (NICE) between January 2011 and May 2023. We used Naïve Bayes, Lasso, and Support Vector Machine models in a binary text classification task (classifying paragraphs as either referencing uncertainty in the evidence base or not). To illustrate the results, we tested hypotheses in relation to the appraisal guidance, advanced therapy medicinal product (ATMP) status, disease area, and age group.

**Results:**

The best performing (Lasso) model achieved 83.6 percent classification accuracy (sensitivity = 74.4 percent, specificity = 92.6 percent). Paragraphs classified as referencing uncertainty were significantly more likely to arise in highly specialized technology (HST) appraisals compared to appraisals from the technology appraisal (TA) guidance (adjusted odds ratio = 1.44, 95 percent CI 1.09, 1.90, *p* = 0.004). There was no significant association between paragraphs classified as referencing uncertainty and appraisals for ATMPs, non-oncology RDTs, and RDTs indicated for children only or adults and children. These results were robust to the threshold value used for classifying paragraphs but were sensitive to the choice of classification model.

**Conclusion:**

Using supervised learning models for text classification in NICE appraisals for RDTs is feasible, but the results of downstream analyses may be sensitive to the choice of classification model.

## Introduction

Health technology assessment (HTA) decision-making processes aim to determine the value of health technologies for a healthcare system (1), and are often used to inform guidance on whether they should be paid for from public funds (2). During these processes, typically a wide variety of text documents are produced and are often made available, on an almost daily basis, on the websites of HTA agencies. For example, for one appraisal by the National Institute for Health and Care Excellence (NICE) this can include the final scope, company evidence submissions, assessments by an independent research group, final appraisal documents, and if applicable, details about managed access agreements. Taken together, these documents usually amount to several hundreds of pages of information that is structured differently depending on the time of publication and the specific appraisal guidance. To retrieve information and analyze HTA appraisal documentation, researchers investigating HTA decision-making processes usually employ manual data extraction and text analysis approaches (3–5).

However, computational text analysis methods using automated approaches for information extraction and classification of large-scale collections of texts are available and are increasingly applied in the social sciences (6). These methods have proliferated in recent years, mostly due to increases in computing power, rapidly evolving advances in natural language processing and machine learning, and the increasing availability of digitized text data (7). Against this background, several reasons motivate the adoption of automated text analysis techniques to analyze HTA appraisals. First, manual text analysis is usually cost- and time-intensive for a large number of complex documents, and automated methods can reduce this burden (8). Second, while manual approaches usually have high validity, they are also likely to have low reliability compared to automated approaches, as manual coding is typically marked by significant inter-coder variability. By contrast, automated approaches promise high reliability but are frequently associated with validity issues (9;10). This motivates the exploration of methods to improve and quantify the validity of automated approaches. Third, automated text analysis techniques have rarely been applied to analyze published HTA appraisals, with only one study (11) known to us at the time of writing. Therefore, the objective of this paper is to explore the application, feasibility, and validity of automated text analysis techniques in the context of HTA appraisals.

To do so, we focus on one approach to automated text analysis – supervised learning methods – and use this approach to classify text in HTA appraisals for rare disease treatments (RDTs) published by NICE. We use a binary text classification task to classify paragraphs as either referencing uncertainty in the evidence base or not. We chose this text classification task because RDTs are typically associated with considerable uncertainty in the clinical and economic evidence (12;13). Thus, understanding how this uncertainty is captured in different RDT appraisals is important. We chose NICE appraisals because NICE explicitly acknowledges the challenges in the generation and interpretation of evidence for innovative and complex health technologies, those targeting rare diseases and pediatric conditions, accepting a higher degree of uncertainty in these contexts (14). Additionally, NICE appraisals are available in an accessible digital format through the NICE syndication application programing interface (API) which facilitates data preprocessing. To illustrate the results of the classification task, we also test several hypotheses about differences between RDT appraisals in relation to the appraisal guidance, advanced therapy medicinal product (ATMP) status, disease area, and age group.

## Methods

### Appraisal Selection

The source documents for this analysis were the final appraisal determination or evaluation documents (FAD/FED) for RDTs appraised under NICE’s technology appraisal (TA) guidance and highly specialized technology (HST) appraisal guidance. We chose these documents because they provide the reasons why a particular reimbursement recommendation was made and as such insights into how evidentiary uncertainty was considered in the appraisals.

Because the NICE guidance website does not allow for filtering of RDTs specifically, we identified the selection of RDT appraisals using the Orphan Register of the Medicines & Healthcare products Regulatory Agency (MHRA) (15). If an appraisal was available for a drug registered as an orphan product in the MHRA Orphan Register, we considered it to be an RDT appraisal. We considered all RDT appraisals published by NICE between January 2011 and the beginning of May 2023 for inclusion in the selection. After excluding terminated appraisals, withdrawn appraisals, appraisals that were replaced by updated guidance, and multiple TAs, we included 94 appraisals in the final dataset (Supplementary Material 1). The main rationale for choosing January 2011 to May 2023 as the time frame for our analysis was to create a relatively large selection of the most recent RDT appraisals. During this time, two regulatory changes took place that have some bearing on how uncertainty might have been discussed in FADs/FEDs, namely the introduction of the HST guidance (in 2013) and the revision of the Cancer Drugs Fund (CDF) (in 2016). While the introduction of the HST guidance changed the appraisal process for some RDTs more so than the way uncertainty was discussed, the revision of the CDF has potentially led to greater description and scrutiny of uncertainty in the FADs/FEDs. However, only five appraisals recommended the RDT for use in the CDF, and only six appraisals were conducted following additional data collection in the CDF. Therefore, we do not believe that these regulatory changes have significantly impacted our analysis.

### Text Acquisition and Preprocessing

We downloaded appraisal files through the NICE syndication API. We performed initial parsing of the files to extract all text and headings in paragraph form, except headers and footers, in Python 3.11.2 (16), using the *beautifulsoup4* library (17). For the classification process, we chose paragraphs as the unit of analysis to reflect the structure of the appraisal documents. We considered any bullet points as being part of the preceding paragraph. We performed all subsequent preprocessing and analysis steps in R 4.2.2 (18). Using *tidyverse* packages (19), we removed several text elements because they were not considered relevant to the classification of uncertainty, including all sections following the appraisal conclusion, tables with the results of clinical studies, headings and section numbers in summary tables, and all other headings except subheadings in TA appraisals as they often provide short summaries. As these elements were unlikely to be predictive of the uncertainty classifications, by removing them, we aimed to reduce the variance of the classifiers without introducing bias. After removal of these elements, the dataset included 4958 text observations (henceforth referred to as “paragraphs”). To prepare the dataset for analysis, additional preprocessing included converting textual features into a quantitative document-feature matrix (DFM), in which rows indicate paragraphs and columns indicate text features. Using the *quanteda* package (20), we created several DFMs with different feature selection choices (Supplementary Material 2). Feature selection choices included common data preprocessing techniques, including the removal of punctuation, numbers, symbols, and stop words. In addition, we removed text features that appeared fewer than five times in the dataset, applied word stemming (reducing words to their base word), and included bigrams (sequences of two adjacent text features).

### Text Classification

Supervised learning methods classify text into pre-determined categories by learning the association between text features (usually words) and categories on the basis of a sample of human-annotated training data (8). They require a random sample of the dataset to be manually coded into different categories of interest. This coded proportion of the dataset is used to train a chosen classifier and also to validate the performance of the classifier (see below). Although there are many different algorithms one can use to conduct supervised learning, these models all aim to learn how different categories of documents use words at different rates, and then use that information to predict the categories of uncoded documents. In this study, we manually coded paragraphs from NICE appraisals for RDTs to train and test three models: Naïve Bayes (21), Lasso regression (22), and Support Vector Machines (SVM) (23). These are frequently used models for text classification, tend to have good classification performance, and are simpler and computationally cheaper to implement than some more sophisticated supervised learning approaches (24–27). We used the *caret* (28), *glmnet* (29), *e1017* (30), and *quanteda.textmodels* (31) packages to estimate the models.

For the manual coding, we classified paragraphs into either of the following two categories: (i) paragraphs with reference to uncertainty in the evidence base (henceforth referred to as “uncertainty paragraphs”) and (ii) paragraphs without reference to uncertainty in the evidence base. We coded paragraphs as uncertainty paragraphs when references to types of uncertainty (heterogeneity, stochastic, parameter, structural, and methodological uncertainty) (32) or sources of uncertainty (transparency, methods, imprecision, bias, or unavailability) (33) were made. In addition, we coded paragraphs describing scenario or exploratory analyses, the committee’s preferred assumptions or data sources, data that will be collected, or further research considered useful, as uncertainty paragraphs. We manually coded a random sample of 15 percent of paragraphs (stratified by HST/TA guidance status) according to the classification criteria. A second researcher repeated the manual coding process for a random sample (20 percent) of the coded paragraphs to validate the manual coding approach. Following the benchmark scale suggested by Landis and Koch (34), there was substantial agreement (kappa statistic: 0.80) between researchers. Any discrepancies were discussed until reaching consensus.

Using cross-validation (35;36), we estimated the performance of the three classifiers for each of the different DFMs. In cross-validation, the training set is randomly split into *k* folds of approximately equal size. The classifier is then trained on *k* − 1 folds and evaluated on the *k*th fold, the hold-out fold, to assess performance. Cross-validation therefore allows the researcher to evaluate the performance of the classification procedure on out-of-sample data that is not used in the training of the model. The estimated performance for each of the *k* hold-out folds is then averaged to obtain an overall estimate of performance. For this study, we repeated this process 10 times (*k* = 10) in line with the established literature (27).

We reported and compared averaged *k-*fold cross-validation performance (classification accuracy, sensitivity, and specificity) for all models using a base case threshold of 0.5 (if the probability of referencing uncertainty of a paragraph was equal to or higher than 0.5, we classified the paragraph as an uncertainty paragraph). We selected the model with the highest accuracy performance – the Lasso model estimated on the stemmed DFM – as the best performing text classification model and subsequently used it for the base case analyses. However, there were not large performance differences between the models. Based on the predicted probabilities of the best performing text classification model, we assigned all paragraphs to a binary category (uncertainty paragraph vs. no uncertainty paragraph) using the base case threshold. We chose a threshold of 0.5 because our sensitivity analyses showed that it provided higher out-of-sample aggregate accuracy scores for the Lasso model than the use of other thresholds. Additionally, this decision rule is intuitive because it means assigning a paragraph to the category for which the model suggests the posterior probability of classification is greatest.

### Statistical Analyses

Following the classification of paragraphs with the best performing text classification model (Lasso), we investigate five hypotheses using univariable and multivariable binary logistic regression models, where our dependent variable was 1 if a paragraph was an uncertainty paragraph and 0 otherwise. We provide an overview of all covariates in Supplementary Material 3. We reported clustered standard errors at the appraisal level. To adjust for multiple hypothesis testing, we applied the Bonferroni correction to confidence intervals and *p*-values for all logistic regression models.

### Hypotheses

We tested the following hypotheses to explain differences between RDT appraisals in relation to uncertainty.

(1) The first hypothesis is that uncertainty paragraphs are more likely to arise in appraisals for RDTs appraised under the HST appraisal guidance compared to the TA guidance. This is because under the HST appraisal guidance typically higher incremental cost-effectiveness ratios (ICERs) and increased uncertainty are accepted (37;38).

(2) The second hypothesis is that uncertainty paragraphs are more likely to appear in appraisals for RDTs that are classified as ATMPs by the European Medicines Agency compared to non-ATMPs. This is because of uncertainties and limitations in the clinical data and economic evaluations of ATMPs, including trial follow-up periods that are usually too short to observe long-term treatment effects, small sample sizes, and single-arm studies (39;40).

(3) Oncology research is well-funded, witnessing a rise in the number of scientific publications (41), and an increase (of 56 percent) in the number of trials between 2016 and 2021, many of which focus on rare cancer indications (42). As such, much less knowledge may exist about non-oncological rare conditions potentially increasing the level of uncertainty in these appraisals. The third hypothesis is that uncertainty paragraphs are more likely to appear in appraisals for RDTs that are indicated for non-oncological conditions compared to oncological conditions.

(4 and 5) Given that many rare diseases affect children, there is also growing interest by the pharmaceutical industry to develop therapeutic options for pediatric populations. However, both conducting clinical studies and performing HTA evaluations for pediatric populations remains challenging, mostly due to issues related to appropriate study designs, outcome measurement, patient recruitment, and ethical considerations (43;44). The fourth hypothesis is that uncertainty paragraphs are more likely to arise in appraisals for RDTs that are indicated for children only compared to adults only. Similarly, the fifth hypothesis is that uncertainty paragraphs are more likely to arise in appraisals for RDTs that are indicated for both adults and children compared to adults only.

### Sensitivity Analyses

We conducted different sensitivity analyses to check the robustness of the results. First, we compared overall cross-validation performance estimates for each of the three models (Naïve Bayes, Lasso, and SVM), estimated on the stemmed DFM, across different threshold values for the probability to classify paragraphs as uncertainty paragraphs. Second, we compared the robustness of the multivariable logistic regression results for each of the hypotheses on the choice of threshold value to classify paragraphs as uncertainty paragraphs, generated by the best performing text classification model (Lasso). Third, we compared the multivariable logistic regression results for each of the hypotheses based on the best performing text classification (Lasso) model with the multivariable logistic regression results where paragraphs were classified as uncertainty paragraphs using the SVM and Naïve Bayes models.

## Results

### Descriptive Statistics

The dataset included 71 appraisals from the TA guidance and 23 appraisals from the HST guidance (Table 1). Among all RDT appraisals, 47.87 percent (*n* = 45) were indicated for oncological conditions, 11.70 percent (*n* = 11) were ATMPs, and 8.51 percent (*n* = 8) were indicated for children only.

**Table 1.** Characteristics of analyzed RDT appraisals (2011–2023) and their corresponding paragraphs (stemmed DFM, base case threshold of 0.5)

	Number of appraisals (%)	Number of paragraphs (%)	Number of paragraphs referencing uncertainty (%)
Total	94 (100.00)	4958 (100.00)	1952 (100.00)
Guidance			
TA	71 (75.53)	3756 (75.76)	1375 (70.44)
HST	23 (24.47)	1202 (24.24)	577 (29.56)
ATMP status			
No	83 (88.30)	4408 (88.91)	1710 (87.60)
Yes	11 (11.70)	550 (11.09)	242 (12.40)
Disease area			
Oncology	45 (47.87)	2367 (47.74)	843 (43.19)
Other	49 (52.13)	2591 (52.26)	1109 (56.81)
Age group			
Adults	61 (64.89)	3219 (64.93)	1190 (60.96)
Children	8 (8.51)	361 (7.28)	164 (8.40)
Both	25 (26.60)	1378 (27.79)	598 (30.64)

ATMP, advanced therapy medicinal product; DFM, document-feature matrix; HST, highly specialized technology appraisal guidance; RDT, rare disease treatment; TA, technology appraisal guidance.

### Classifier Performance

The three classifiers produced similar aggregated accuracy performance results across the DFMs ranging between 78.6 and 83.6 percent, suggesting that different feature selection choices only had a marginal impact on aggregate classification performance. The Lasso model estimated using the stemmed DFM demonstrated the highest average cross-validation accuracy and specificity (accuracy = 83.6 percent, sensitivity = 74.4 percent, specificity = 92.6 percent) (Supplementary Material 4). These results indicate that over 8 out of 10 of all paragraphs are correctly classified, approximately 7.5 out of 10 cases of uncertainty paragraphs are correctly classified, and more than 9 out of 10 cases of non-uncertainty paragraphs are correctly classified, justifying the use of the measure in downstream analyses. The predicted probabilities between the three models estimated on the stemmed DFM were positively correlated (Supplementary Material 5).

Face validity checks of the top 10 uncertainty paragraphs confirmed that several types and sources of uncertainty were discussed in each paragraph, including uncertainties in the clinical and economic modeling evidence in relation to model structure, transition probabilities, health utilities, treatment stopping criteria, dosage, administration costs, indirect comparisons, survival benefit, and trial design (Supplementary Material 6).

### Classification Results

**Figure 1.**
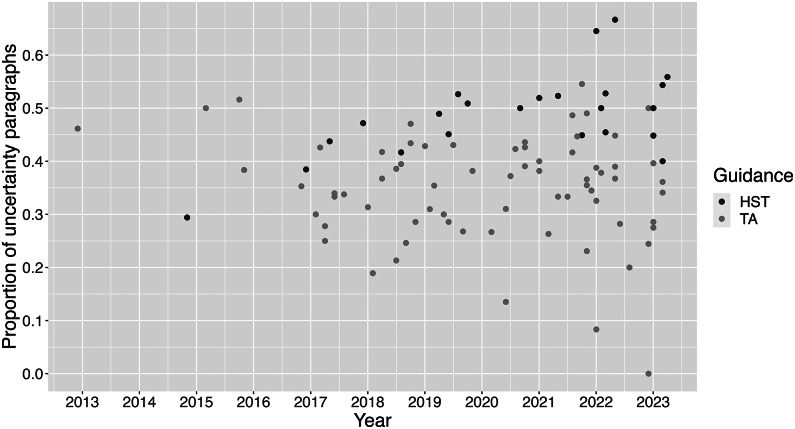
Proportion of uncertainty paragraphs per appraisal over time (stemmed DFM, base case threshold of 0.5). DFM, document-feature matrix; HST, highly specialized technology appraisal guidance; TA, technology appraisal guidance.

Out of a total of 4958 paragraphs, 1952 (39.37 percent) were classified as uncertainty paragraphs (Table 1). There are differences in the proportion of uncertainty paragraphs per appraisal over time although no clear trend appears (Figure 1).

**Figure 2.**
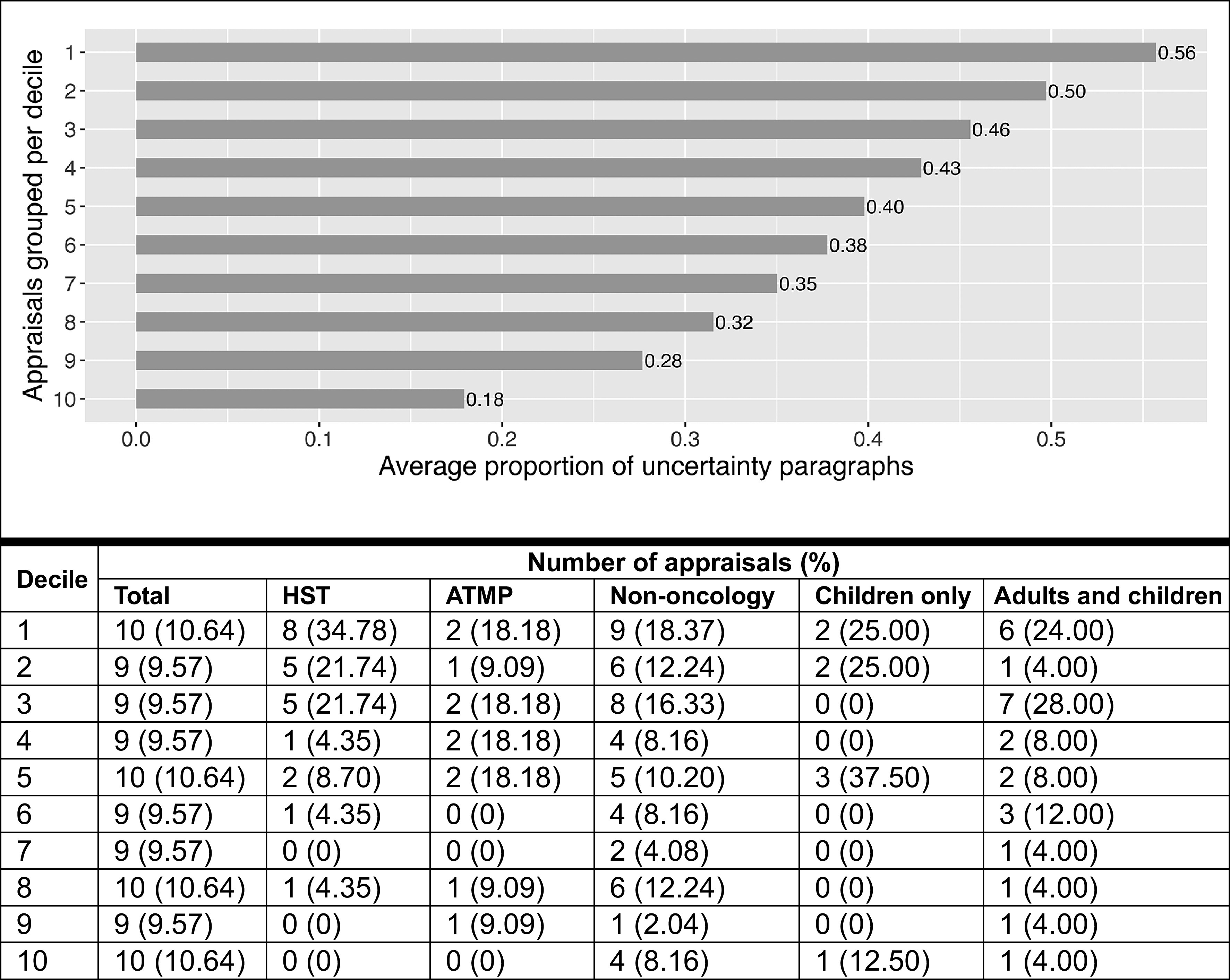
Average proportion of uncertainty paragraphs per decile (stemmed DFM, base case threshold of 0.5). ATMP, advanced therapy medicinal product; DFM, document-feature matrix; HST, highly specialized technology appraisal guidance.

Moreover, grouping appraisals per decile showed that the average proportion of uncertainty paragraphs was 56 percent in the highest decile (1) compared to 18 percent in the lowest decile (10) (Figure 2). Among RDT appraisals in deciles 1–5 (*n* = 47), 44.68 percent (*n* = 21) were appraised under the HST appraisal guidance, 68.09 percent (*n* = 32) were indicated for non-oncological conditions, 19.15 percent (*n* = 9) were ATMPs, 14.89 percent (*n* = 7) were indicated for children only, and 38.30 percent (*n* = 18) for both adults and children.

### Statistical Analyses

Table 2 reports the adjusted odds ratios (AOR) of the multivariable logistic regression model with uncertainty paragraphs as the dependent variable. The model showed that uncertainty paragraphs were significantly more likely to appear in HST appraisals compared to TA appraisals (AOR = 1.44, 95 percent CI 1.09, 1.90, *p*-value = 0.004). There was no significant association between uncertainty paragraphs and appraisals for RDTs classified as ATMPs compared to non-ATMPs, appraisals for RDTs non-oncological conditions compared to oncological conditions, and appraisals for RDTs indicated for children only or both adults and children compared to adults only. Except for ATMP status, all univariable logistic regression models modeling the covariates individually showed significant associations and are reported in Supplementary Material 7.

**Table 2.** Multivariable logistic regression model with uncertainty paragraphs as dependent variable (stemmed DFM, base case threshold of 0.5, *N* = 4958)

Covariate	Level	AOR	95% CI*	Clustered SE	*p*-value*
Guidance					
	TA^a^	–	–	–	–
	HST	1.44	1.09, 1.90	0.108	0.004
ATMP status					
	No^a^	–	–	–	–
	Yes	1.14	0.90, 1.45	0.092	0.753
Disease area					
	Oncology^a^	–	–	–	–
	Other	1.12	0.86, 1.47	0.105	1.000
Age group					
	Adults^a^	–	–	–	–
	Children	1.04	0.77, 1.39	0.114	1.000
	Both	1.05	0.81, 1.35	0.098	1.000

*Note:* Model adjusted for guidance type, ATMP status, disease area, and age group.

a Reference level.

* Bonferroni-adjusted confidence intervals and *p*-values (number of hypotheses = 5).

AOR, adjusted odds ratio; ATMP, advanced therapy medicinal product; CI, confidence interval; DFM, document-feature matrix; HST, highly specialized technology appraisal guidance; SE, standard error; TA, technology appraisal guidance.

### Sensitivity Analyses

Cross-validation accuracy scores of all three classifiers varied for different threshold values, but the best performing text classification model (Lasso), estimated on the stemmed DFM, achieved its highest accuracy score at a threshold of 0.5, supporting our approach of using the value of 0.5 in our base case analyses (Supplementary Material 8). Moreover, the results of the multivariable regression analysis based on the base case (Lasso) model were robust to different threshold values for the probability to classify paragraphs as uncertainty paragraphs (Supplementary Material 9).

Comparing the performance of the Naïve Bayes and SVM model against the base case (Lasso) model showed that the proportion of uncertainty paragraphs was different for each model (Lasso: 39.37 percent, Naïve Bayes = 57.93 percent, SVM = 42.90 percent). Moreover, the results of the multivariable regression model were sensitive to the choice of the classifier. The base case model showed a significant association between uncertainty paragraphs and HST appraisals (AOR = 1.44, 95 percent CI 1.09, 1.90, *p* = 0.004). Estimates based on the classification from the SVM model are of a comparable magnitude, though are not statistically significant (AOR = 1.38, 95 percent CI 0.92, 2.09, *p* = 0.215). Estimates based on the Naïve Bayes classification differ in both sign and significance (AOR = 0.82, 95 percent CI 0.58, 1.17, *p* = 0.750). Nonsignificant associations between the dependent variable and ATMP status, disease area, and age group are robust across all three classification models (Supplementary Material 10).

## Discussion

This study applied a supervised learning approach to perform binary text classification of NICE appraisals for RDTs using three classifiers (Naïve Bayes, Lasso, and SVM). The Lasso model using a stemmed DFM demonstrated the highest average cross-validation accuracy score (83.6 percent). Therefore, it was chosen as the best performing text classification model and used for base case analyses. Face validity checks of the top uncertainty paragraphs predicted by the classifier confirmed that different types and sources of uncertainty were described in each paragraph. This is similar to de Folter et al. (11) who show that uncertainty is typically associated with many decision factors, including in the clinical effectiveness evidence, health utility estimates, economic modeling, ICER estimates, and comparators among others. Overall, our analyses demonstrated that applying supervised text classification methods to HTA appraisals can be done and is feasible for a binary text classification task in relation to uncertainty in NICE appraisals for RDTs.

Overall, the base case analysis showed that uncertainty paragraphs were significantly more likely to appear in HST appraisals compared to TA appraisals. This result proved to be robust in sensitivity analyses using different thresholds for the probability of classifying a paragraph as uncertainty paragraph or not. This is also intuitive and consistent with the purpose of the HST guidance, which targets the evaluation of drugs for very severe and rare diseases for which there is typically no adequate treatment alternative (38). However, the sensitivity analyses demonstrated that when regression models were estimated on the basis of classifications from the Naïve Bayes or SVM models, this significant association disappeared. This result might be attributed to the small number of appraisals (*n* = 94) or other potentially relevant process- and drug-related factors which this analysis did not account for. Therefore, the results of the hypothesis tests illustrating the results of the classification task should be interpreted with caution.

The base case analysis also showed no evidence for an association of uncertainty paragraphs and ATMP appraisals, non-oncology appraisals, and appraisals indicated for children only or indicated for both adults and children. These results were robust across the choice of classifier and raise the question to what extent the challenges in the generation of robust clinical and economic evidence and the uncertainties surrounding RDTs, which classify as ATMPs, are indicated for non-oncological conditions or children are distinctive compared to RDTs without these characteristics. The high levels of uncertainty generally associated with RDTs may have contributed to the lack of support for these hypotheses. Nonetheless, this study demonstrates that investigating different hypotheses is much more feasible if approaches for data classification and extraction are automated.

This study has a few limitations. First, the best performing text classification model is not completely accurate due to its accuracy performance of 83.6 percent. Thus, some paragraphs probably have been incorrectly classified. Second, the manual coding of paragraphs to train the classifiers was done by one researcher only, increasing the risk of bias. However, the validation of a subset of manually coded paragraphs by a second researcher helped to reduce this bias. Third, there might be a difference in the nature and number of RDT appraisals included in the dataset depending on the inclusion and exclusion criteria. For example, this study excluded appraisals which were replaced by updated guidance because they were no longer available in the NICE guidance database. Some updated appraisals may systematically differ in terms of discussing uncertainty which we note as a limitation.

## Conclusion

This study used supervised learning models to classify text in relation to uncertainty in NICE appraisals for RDTs. The findings confirm the feasibility and validity of this approach and provide some insights into the characteristics of appraisals for RDTs evaluated by NICE. However, regression results based on the classification should be interpreted with caution due to sensitivity to the choice of classification model. While our study has illustrated the potential of using automated text classification techniques for analyzing HTA appraisals, further work is required to demonstrate the robustness of this method across a wider selection of drug appraisals. We hope that these findings motivate other researchers to explore automated text analysis techniques to further examine differences in HTA appraisals and processes. For example, applying supervised learning approaches for text classification comparatively to both appraisals for RDTs and non-RDTs could be an exciting opportunity for further research.

## Supporting information

Wiedmann et al. supplementary material Wiedmann et al. supplementary material

## Supplementary material

The supplementary material for this article can be found at https://doi.org/10.1017/S0266462323002805.
