# Supplementary material for: Using automated text classification to explore uncertainty in NICE appraisals for drugs for rare diseases
Source: Int J Technol Assess Health Care. 2024 Jan 5;40(1):e5. doi: 10.1017/S0266462323002805 (PMC10859832; doi:10.1017/S0266462323002805)
Supplement: Wiedmann et al. supplementary material [file S0266462323002805sup001.pdf]

<u>Using automated text classification to explore uncertainty in NICE appraisals for drugs for rare diseases</u>

Supplementary material

Table of Contents

Supplement 1 – appraisal selection 2

Supplement 2 – feature selection choices 3

Supplement 3 - covariates 4

Supplement 4 – classifier performance results 5

Supplement 5 – correlations and distributions 6

Supplement 6 – top 10 uncertainty paragraphs 7

Supplement 7 – univariable regression results 13

Supplement 8 – classification performance across thresholds 14

Supplement 9 – multivariable regression results across thresholds 15

Supplement 10 – multivariable regression results across models 16

Supplement 1 – appraisal selection

Table 1: Appraisal selection

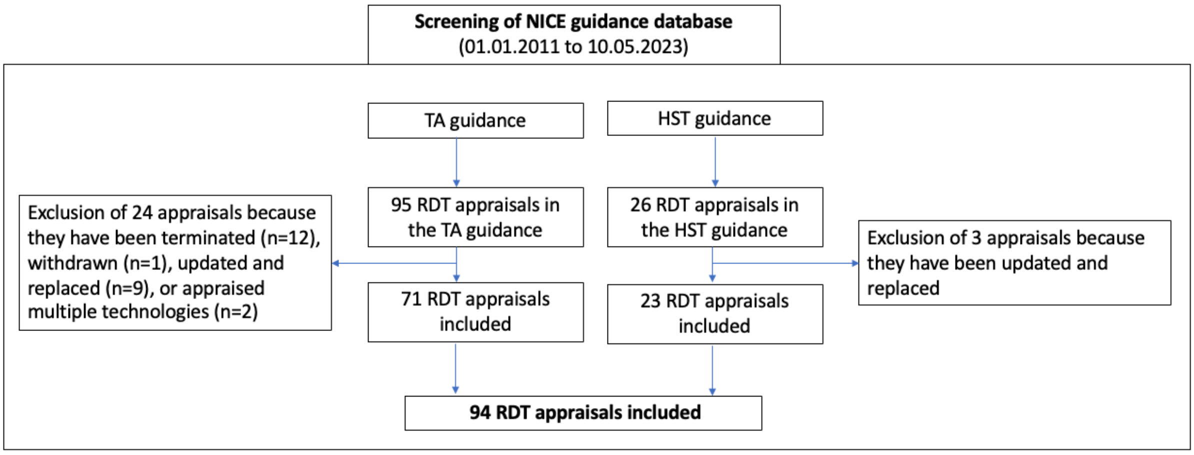

HST = Highly specialized technology appraisal guidance, NICE = National Institute for Health and Care Excellence, TA = Technology appraisal guidance, RDT = rare disease treatment

Supplement 2 – feature selection choices

Table 2: Overview of feature selection choices

**Document-feature matrix (DFM)**	**Feature selection choices**
Original DFM	Removal of punctuation, numbers, symbols, and stop words<br>Unigrams
Raw DFM	Nothing removed
Stemmed DFM	Removal of punctuation, numbers, symbols, and stop words<br>Unigrams<br>Word stemming (reduction of text features to their stem)
Trimmed DFM	Removal of punctuation, numbers, symbols, and stop words<br>Unigrams<br>Removal of tokens that appear fewer than five times in the corpus
N-grams DFM	Removal of punctuation, numbers, symbols, and stop words<br>Unigrams and bigrams

DFM = document-feature matrix

Supplement 3- covariates

Table 3: Covariates

Variable name	Description	Coding
Guidance	Whether the RDT was appraised under the technology appraisal (TA) or the highly specialized technology (HST) appraisal guidance.	0. TA<br>1. HST
ATMP	Whether the RDT was classified as an advanced therapy medicinal product (ATMP) by the European Medicines Agency (EMA).	0. No<br>1. Yes
Disease area	The disease area of the RDT based on its indication.	0. Oncological condition<br>1. Non-oncological condition
Age group	Whether the RDT is indicated for adults (>=18 years), children (< 18 years) or both.	1. Adults<br>2. Children<br>3. Both

ATMP = advanced therapy medicinal product, RDT = rare disease treatment

Supplement 4 – classifier performance results

Table 4: Classifier performance results (base case threshold of 0.5)

Models	Accuracy	Sensitivity	Specificity
**Stemmed DFM**			
Lasso	0.836	0.744	0.926
Naïve Bayes	0.796	0.890	0.701
SVM	0.808	0.714	0.897
**Raw DFM**			
Lasso	0.824	0.744	0.902
Naïve Bayes	0.811	0.898	0.720
SVM	0.793	0.691	0.890
**Original DFM**			
Lasso	0.831	0.747	0.914
Naïve Bayes	0.807	0.896	0.716
SVM	0.796	0.712	0.876
**Trimmed DFM**			
Lasso	0.827	0.733	0.920
Naïve Bayes	0.810	0.853	0.764
SVM	0.786	0.710	0.859
**N-grams DFM**			
Lasso	0.821	0.715	0.924
Naïve Bayes	0.800	0.883	0.714
SVM	0.794	0.716	0.866

DFM = document-feature matrix, SVM = Support Vector Machines
The model highlighted in yellow was chosen as the best performing text classification model.

Supplement 5 – correlations and distributions

Table 5: Correlations between predicted probabilities (stemmed DFM)

	Naïve Bayes	Lasso	Support Vector Machines
**Naïve Bayes**	1		
**Lasso**	0.627	1	
**Support Vector Machines**	0.539	0.740	1

Figure 1: Correlations between predicted probabilities (stemmed DFM)

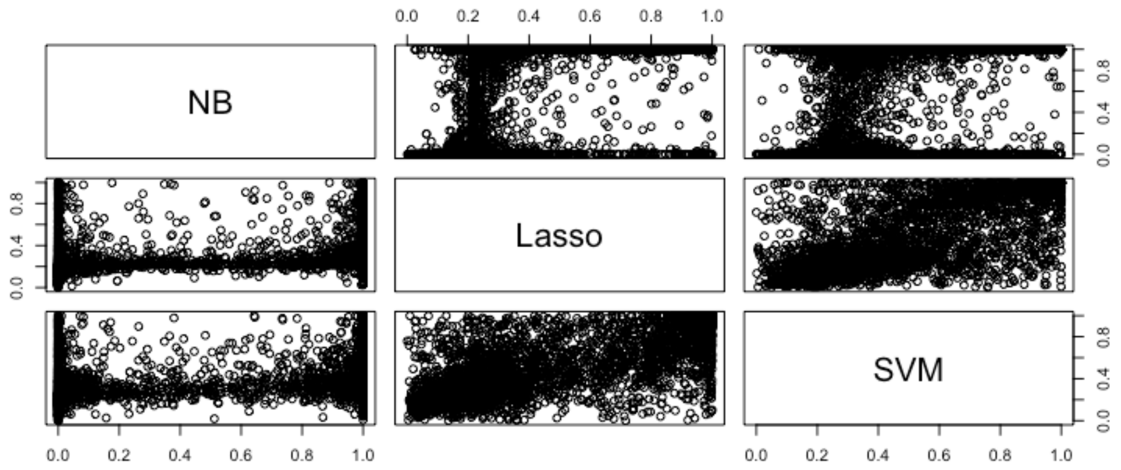

NB = Naïve Bayes, SVM = Support Vector Machines

Figure 2: Distributions of predicted probabilities of each classifier

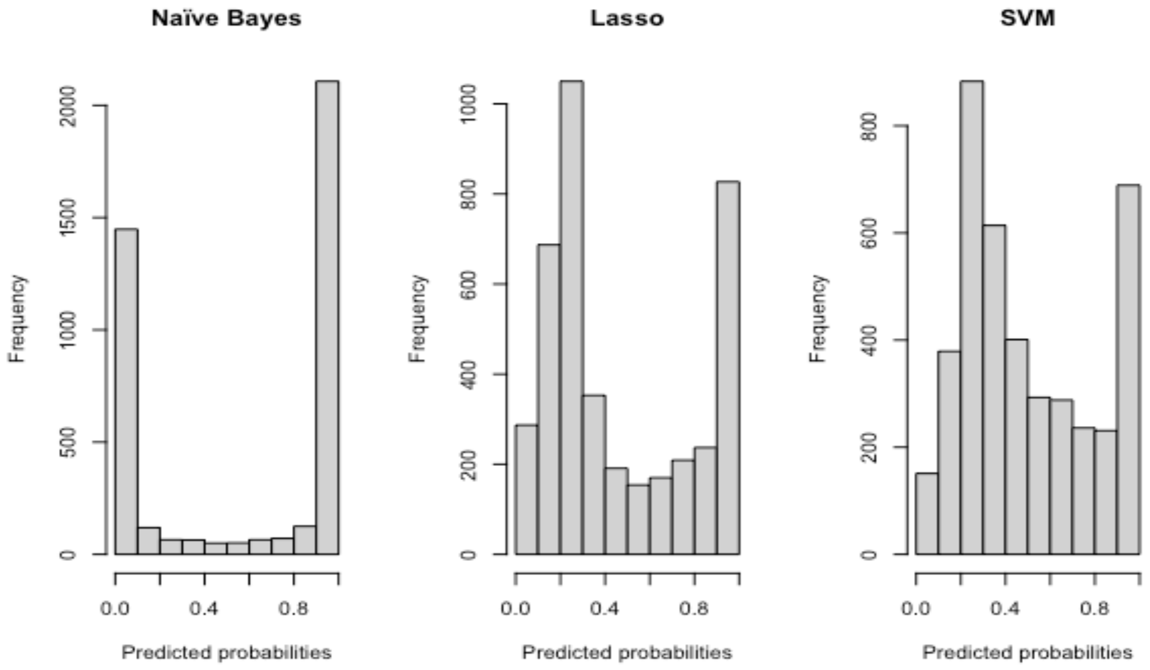

SVM = Support Vector Machines

Supplement 6 – top 10 uncertainty paragraphs

Table 6: Paragraphs with the highest predicted probabilities of referencing uncertainty (Top 10) (Lasso model, stemmed DFM, base case threshold of 0.5)

Top 1 (text1039)	"3.7 The marketing authorisation for tafamidis does not specify starting and stopping rules for tafamidis based on the NYHA classification system. The company highlighted that NYHA classifications have been incorporated in previous NICE recommendations to define populations eligible for treatment with heart failure therapies. The committee noted that the marketing authorisation states that tafamidis should be 'started as early as possible in the disease course when the clinical benefit on disease progression could be more evident. Conversely, when amyloid-related cardiac damage is more advanced, such as in NYHA class 3, the decision to start or maintain treatment should be taken at the discretion of a physician knowledgeable in the management of patients with amyloidosis or cardiomyopathy'. The committee recalled that NYHA class 1 means that people can do ordinary physical activity (see section 3.6). It considered if tafamidis would be used for people who are easily able to do the activities of daily living (no functional limitations). The clinical experts explained that they would have reservations about offering treatment to people whose disease is classed as NYHA 1 because they have no functional limitations and might not benefit from treatment. At consultation, the company highlighted that this contradicted tafamidis' marketing authorisation, which states that treatment should be started as soon as possible. The company proposed a stopping rule in which people would stop tafamidis if their disease progressed to NYHA class 4. It explained that there was limited evidence to support using tafamidis in people whose disease was NYHA class 4, who had severe heart failure symptoms, because they were excluded from the ATTR-ACT pivotal trial. Also, the company highlighted that its proposed stopping rule reflected treatment stopping in ATTR-ACT, in which most people stopped tafamidis quickly after progressing to NYHA class 4. It also noted that because tafamidis does not improve symptoms caused by ATTR-CM it would be clinically appropriate to stop treatment when a person's disease is classed as NYHA 4. A clinical expert noted that stopping treatments when the disease progresses to NYHA class 4 was common because at this stage people are very unwell. They explained that people would be unable to travel for treatment, so treatment would likely be stopped and best supportive care offered. Comments from the patient organisation supported this view, stating that making decisions about stopping treatment in advanced disease stages were not uncommon. Conversely, 2 of the clinical experts noted that it would be challenging to stop treatment when disease progressed to NYHA class 4 because no alternative treatments were available. These 2 clinical experts also explained that people's disease often varies between NYHA class 3 and 4 and that this was typical of ATTR-CM. They noted that some people whose disease was classed as NYHA 4 could improve, so could change to NYHA class 3 or better. The ERG noted that improvements shown by changes in NYHA class were also seen in ATTR-ACT. The committee recalled that the company's proposed stopping rule was not specified in tafamidis' marketing authorisation. It agreed, that on balance, it would be difficult for clinicians to implement a stopping rule for tafamidis. This was because the disease can often vary between NYHA class 3 and 4 and the lack of alternative treatments for NYHA class 4 disease meant people would likely prefer to keep taking tafamidis. The committee concluded that using the NYHA classification alone to accurately define the population who were eligible to have tafamidis had limitations. So, it also concluded that it would not consider starting and stopping rules for tafamidis based on the NYHA classification system in its decision making."
Top 2 (text3367)	"5.11 The committee discussed the company's cost–consequence model and the assumptions on which it was based. It noted that the model structure was complex but reflected the important health states. The committee discussed the key assumptions included in the company's economic model: In the absence of direct evidence comparing eliglustat with ERT in patients who had not previously had treatment, the company assumed that eliglustat and ERT have equal efficacy in such patients. The ERG stated that evidence from the ENCORE trial would have been more appropriate. Following consultation, the company stated that the mean treatment duration with ERT before entering ENCORE was about 10 years, so these data could not be generalised to people who had not previously had treatment. The company stated that its assumption of equivalence was supported by an indirect comparison (Ibrahim et al., 2016) on the basis of which the European Medicines Agency's Committee for Medicinal Products for Human Use stated that comparable results can be expected. The ERG agreed that using data from ENCORE was not ideal, but considered that it was superior to the company's approach. The company used data from ENGAGE to estimate transition probabilities for patients having eliglustat, and applied these to both treatment arms in the first cycle of the model. The ERG stated that this did not capture any potential differences between eliglustat and ERT. The committee agreed that both approaches had limitations. It heard that, because these transition probabilities were applied to the first cycle only, it had a very small impact on the results. The company assumed long-term equivalence of eliglustat and ERT, and the ERG highlighted that this had a considerable impact on estimated incremental quality-adjusted life years (QALYs). The committee agreed with the ERG that non-inferiority was not the same as equivalence, and that non-inferiority in the short term does not imply non-inferiority in the long term. The committee considered the 4-year data presented by the company following consultation (see section 5.8) and also noted that the company presented varied approaches to transition within the model, resulting in a negligible impact on total QALYs gained. The ERG, however, clarified that the

	assumption of long-term equivalence was not underpinned by how transition probabilities are calculated, but by using the same probabilities in the long term across both arms of the model. The committee maintained that there was uncertainty around the assumption of equivalence in the long term. The dosage of ERT used in the model was 42.4 U/kg every 2 weeks, based on the mean dose of imiglucerase patients had in the ENCORE study. The committee recalled (see section 5.4) that a dose of between 15 U/kg and 30 U/kg was considered most reflective of clinical practice. The committee was aware that the dose of ERT was a key driver of costs and that the ERG had explored the impact of including a dose of 25 U/kg. The committee considered that the ERG exploratory analysis that included a dose of 25 U/kg was appropriate. Following consultation, the company stated that real world weight should also be factored into estimating the total administered dose (see section 4.58). The ERG clarified that that dose of ERT in the ERG analyses was obtained from English prescribing data reporting average units per month, so the average weight in the model was not relevant. However, the ERG presented exploratory analyses using estimates based on real world weight. The company assumed that the mortality risk does not increase with disease severity. The committee considered that this was an unrealistic assumption. It noted that the ERG explored the impact of increased mortality risk for patients in the 'marked' and 'severe' health states. The company assumed that there are no administration costs associated with eliglustat because it is an oral therapy. The ERG explored including a monthly dispensary cost for eliglustat but, following consultation, the company stated that eliglustat could be dispensed less frequently. The committee agreed with the ERG that there was uncertainty around the frequency and, because this had a minor impact on the results, the ERG's approach of including a monthly dispensary cost was pragmatic. The ERG highlighted that the administration costs for ERT were likely to be overestimated in the company's model because they were higher than the costs of hospital administration. The company stated that this would depend on the perspective of the costing analysis, but the ERG confirmed that all data available supported lower costs for home administration. The committee agreed that the ERG's exploration assuming equal cost was appropriate, and potentially overestimates the cost of ERT. The committee considered that these reflected important uncertainties in the model, but was satisfied that the ERG had presented results based on assumptions suitable for decision-making."
Top 3 (text272)	"3.9 The company originally used 20-week data from SOLSTICE to model CMV recurrences up to 52 weeks, meaning its stage 1 Markov model had a duration of 52 weeks. But based on the OTUS data (which provided evidence for multiple recurrences over a longer time), the company increased the duration of the stage 1 model to 78 weeks. The ERG was unclear about the company's reasoning for using 78 weeks. The company explained that OTUS data in the SOT population provided evidence that would allow the stage 1 model to be extended beyond 78 weeks, but had applied 78 weeks as a pragmatic option because of heterogeneity in the treatment pathway at longer time horizons and to mitigate uncertainty. The ERG highlighted there were few third (or further) recurrences in OTUS and so to model further recurrences the company had to use the risk of second recurrence from OTUS (see section 3.8). This created uncertainty in the modelling. The ERG thought that the duration of the stage 1 Markov model should reflect the time frame over which the first and second recurrences happened in OTUS (39.2 weeks) because the data for this was robust. It included this assumption in its base case. The committee recognised there was some uncertainty around the appropriate duration of the stage 1 Markov model. But it considered that if OTUS was used as the main source of data for the IAT arm of the model, the stage 1 Markov model should accurately reflect the time to last recurrence in OTUS. The committee agreed at the first meeting that the stage 1 Markov model should align with the duration of time that CMV recurrences can be accurately modelled. It specified that more than 2 CMV recurrences should be modelled, with the risk of recurrence decreasing as the number of recurrences increases, if data was available to model this. In the absence of robust data, the stage 1 Markov model should be restricted to 39.2 weeks and 2 CMV recurrences, and scenario analyses should be done to show the potential impact of further CMV recurrences, with a stage 1 duration of between 39.2 and 78 weeks. In response to consultation, the company accepted the committee's preference, and updated its base case to restrict the stage 1 Markov model to 39.2 weeks and 2 CMV recurrences. The company commented that the OTUS data was a robust source for modelling recurrences over time and that including a maximum of 2 recurrences was conservative. The committee noted that the company had not provided any scenario analyses showing the potential impact of more than 2 CMV recurrences with a stage 1 duration of between 39.2 and 78 weeks, as requested at the first meeting. The ERG was satisfied that the company had updated the model correctly. The committee concluded that the company's updated model was suitable for decision making."
Top 4 (text418)	"3.9 No trials directly compared fenfluramine with cannabidiol plus clobazam. So the company did a network meta-analysis to assess the effectiveness of different dosages of fenfluramine (Study 1: 0.2 mg/kg/day and 0.7 mg/kg/day; Study 1504: 0.4 mg/kg/day) and cannabidiol plus clobazam (10 mg/kg/day and 20 mg/kg/day plus clobazam) relative to placebo. The network meta-analysis was done for both the primary and secondary outcomes of Study 1 and Study 1504. The ERG noted there were differences in the use of standard care drugs including clobazam across trials. The network meta-analysis assessed percentage change from baseline in convulsive seizure frequency in 28 days compared with placebo, which was the primary end point of Study 1 and Study 1504 and informed the economic model. The ERG noted that, while the results showed that all doses of fenfluramine and cannabidiol plus clobazam were more effective than placebo in reducing convulsive seizure frequency per 28 days, there was no difference between fenfluramine and cannabidiol plus clobazam in this analysis. During the first meeting, the committee noted that this analysis did not show a

	difference between fenfluramine and cannabidiol plus clobazam. It also noted that it would prefer to see the absolute changes from baseline associated with different dosages of fenfluramine and cannabidiol plus clobazam. During the consultation, the company explained that data for absolute changes from baseline for cannabidiol plus clobazam is not publicly available, so it was not able to do this analysis. The company instead presented an indirect treatment comparison between fenfluramine, cannabidiol, and placebo on the outcome of percentage change from baseline in convulsive seizure frequency over 28 days using the Bucher method. This additional analysis included data publicly available from 4 trials of cannabidiol plus clobazam (results of the analysis are confidential and cannot be reported here). The committee noted that the comparisons between fenfluramine and different dosages of cannabidiol plus clobazam were mixed but largely favoured fenfluramine. Carer and clinical experts explained during the second meeting that Dravet syndrome is a heterogeneous condition, reflected in the range of seizure frequency and intensity. They said that the differences in results reflected the natural variation in the condition and are expected. The committee noted that the mixed results may be partly because of the small sample sizes in the trials as well as heterogeneity. It questioned why the company did not pool the 2 cannabidiol plus clobazam trials with the same dosing in this additional analysis on the primary end point. The company explained that it was because the committee had requested analysis of the absolute change in convulsive seizure frequency for cannabidiol plus clobazam from baseline compared with fenfluramine during its first meeting, given the uncertainties in the network meta-analysis of the primary end point. However, the company had no access to such data for cannabidiol plus clobazam. So the company did not combine the cannabidiol plus clobazam trials with the same or different dosages, so that the differences in treatment effect on the primary end point between specific dosages of fenfluramine and specific dosages of cannabidiol plus clobazam can be seen. The company also explained that the 2 cannabidiol plus clobazam trials with the maximum recommended dosing for cannabidiol plus clobazam (20 mg/kg/day) reported different treatment effects for the primary end point. The ERG noted that the heterogeneity across trials may be another reason not to pool trials for analysis. The committee acknowledged that, overall, the evidence suggested superiority of fenfluramine compared with cannabidiol plus clobazam but noted that there was high uncertainty given the heterogeneity across trials."
Top 5 (text274)	"3.10 The company had originally modelled survival in the stage 1 Markov model using individual patient data from SOLSTICE to estimate the risk of mortality in the clinically significant CMV and no clinically significant CMV health states. But the ERG noted that the Kaplan–Meier data, which incorporated the difference in CMV events across treatment arms, showed no statistically significant difference in overall mortality between maribavir and IAT (see section 3.4). So this was inconsistent with the company's approach of assuming there was a difference in mortality for clinically significant CMV compared with no clinically significant CMV. At technical engagement, the company reiterated its view that the SOLSTICE data was the most appropriate source. It provided Kaplan–Meier data for time to all-cause mortality from SOLSTICE (adjusted to account for people in the IAT arm crossing over to have rescue treatment). The company did not explain how the adjustment was done, so the ERG could not validate the adjusted survival data. The company considered that its analysis supported using the unadjusted SOLSTICE data in the model. It reiterated its view that SOLSTICE suggested that mortality for maribavir was lower than for IAT, and that this justified using CMV-related mortality risks taken from SOLSTICE in the model. Additionally, the company provided 2 scenario analyses based on OTUS and using published data to inform mortality risks for people who had clinically significant CMV and no clinically significant CMV. The ERG noted that the scenario using the published data (Hakimi et al. [2017] for the SOT population and Camargo et al. [2018] for the HSCT population) did not include populations that fully aligned with either SOLSTICE or the decision problem. At the first meeting, the committee recognised there was a lot of uncertainty in the assumptions for mortality in the stage 1 model, but that SOLSTICE had not shown a survival benefit. It considered that mortality should not differ based on treatment, so there should be no life year gain with maribavir in the model. It agreed that risk of mortality in the stage 1 model should be the same for the maribavir and IAT groups. In response to consultation, the company disagreed with the committee's preference, and maintained that SOLSTICE provided clear evidence of a difference in survival associated with a response to CMV treatment. It provided further evidence including a Kaplan–Meier plot of overall survival by clearance status at week 8 from SOLSTICE, which showed a statistically significant difference in the hazard rate of death between CMV clearance at week 8 (in either treatment group) compared with no CMV clearance. It also provided data from TAK620-5004, a retrospective study collecting follow-up data at 12 months from SOT and HSCT recipients randomised to the maribavir arm in the SOLSTICE study. This data showed numerically lower overall mortality than that seen in published estimates, 12 months after treatment for refractory or resistant CMV after a transplant. The company updated its base case using the published data from Hakimi and Camargo to inform mortality risks for people with clinically significant CMV and no clinically significant CMV. The ERG noted that the risk of mortality associated with CMV was likely higher in the 2 sources used in the company's base case than in SOLSTICE and OTUS, and that the company's base case represented the best-case scenario. The ERG would have preferred this data to come from OTUS had it been available. It agreed with the company that clinically significant CMV is associated with increased mortality, but not with the magnitude modelled by the company. To help with decision making, the ERG provided 2 scenarios: a worst-case scenario with no additional risk of mortality from CMV (aligned with the committee's preference after the first meeting) and a midpoint in which people with CMV were arbitrarily assumed to have twice the risk of mortality than people without CMV. The committee acknowledged that although eliminating clinically significant CMV may reduce mortality, this did

	not mean that maribavir would reduce mortality. It was also aware that assuming a mortality benefit associated with no CMV substantially affected the cost-effectiveness results. The committee accepted that it was very likely that CMV clearance would have an impact on mortality, but the magnitude of the impact was very uncertain. It commented that it was likely that the upper bound of that magnitude was from the published data sources used by the company. The committee concluded that the true value was likely to lie somewhere in between no benefit and that upper bound, and that the company's base case was likely optimistic."
Top 6 (text 1065)	"3.23 The company estimated health state utility values separately for each NYHA class (see section 3.21) and treatment included in the model. It explained that different health state utility values between tafamidis and best supportive care may reflect differences in hospitalisations and adverse events associated with each treatment. The committee recalled that the NYHA classification system was unlikely to be sensitive to changes in ATTR-CM (see section 3.6). The ERG noted that the company modelled substantially different on- and off-treatment utility values in the NYHA class 4 health state. It also explained that estimates of NYHA class 4 utility values were based on very few observations. The company highlighted that the health state utility values were derived from EQ-5D-3L data from the ATTR-ACT pivotal trial and were the most appropriate data for the economic analysis. The ERG noted that in ATTR-ACT quality-of-life data were collected only during the on-treatment period, and that in the trial, most people stopped treatment before their disease progressed to NYHA class 4. The ERG explained that the estimated NYHA class 4 utility value for tafamidis could be affected by informative censoring, because the quality of life of anyone who stopped tafamidis in NYHA class 4 was not captured. To account for this, the ERG's analysis after technical engagement assumed that the estimated best supportive care utility value applied to everyone in the NYHA class 4 health state. After technical engagement the company accepted that it was appropriate to apply the best supportive care utility value in NYHA class 4 and it used this assumption in its revised analysis. The committee agreed that it had concerns about using treatment-dependent health state utility values from relatively few observations and the potential for informative censoring to bias these estimates. It concluded that the treatment-dependent utility values were reasonable in NYHA class 1 to 3, and that the best supportive care utility value should be applied in the NYHA class 4 health state."
Top 7 (text15)	"3.4 Because L-MIND is a single-arm study, indirect treatment comparisons were needed to establish the relative efficacy of tafasitamab plus lenalidomide compared with other treatments. The company used 2 indirect treatment comparison approaches: propensity score matching against RE-MIND2 and matching-adjusted indirect comparisons against published studies. RE-MIND2 was an observational, retrospective cohort study of 3,454 adults with relapsed or refractory diffuse large B-cell lymphoma, including 115 people from the UK. The company used nearest neighbour propensity score matching to balance the cohorts for comparator treatments with L-MIND based on 9 baseline covariates. In the matching-adjusted indirect comparisons the company adjusted the L-MIND population using propensity score weighting to be comparable to the populations in 4 published trials of comparator treatments, which were selected using a systematic literature review and expert input. The company used RE-MIND2 for rituximab with gemcitabine and oxaliplatin and the matching-adjusted indirect comparisons for polatuzumab vedotin with bendamustine and rituximab as well as bendamustine and rituximab. The company chose indirect evidence sources based on alignment to published outcomes. This resulted in RE-MIND2 not being selected for polatuzumab vedotin with bendamustine and rituximab. All the indirect comparisons suggested that tafasitamab with lenalidomide improved progression-free and overall survival compared with the comparators, but this was not always statistically significant. The ERG highlighted that RE-MIND2 consists of pooled individual participant data and is preferred in principle to the intervention population adjustment done in the matching-adjusted indirect comparisons. Adjusting the L-MIND population differently for each comparator treatment population may have led to bias. However, there was uncertainty about the methods used for RE-MIND2 because the baseline characteristics of the tafasitamab with lenalidomide cohort varied depending on the comparator. The ERG suggested that it was unclear what type of treatment effect is estimated in RE-MIND2. The committee concluded that, because of the complexity in the methods used for the indirect treatment comparisons, and the potential biases, the results of the indirect comparisons were very uncertain."
Top 8 (text822)	"3.7 Namuscla is a new formulation of mexiletine that uses different dose measurements to previous off-label use (a 167 mg capsule of Namuscla formulation is equivalent to 200 mg of imported mexiletine). However, all the clinical evidence uses the imported formulation of mexiletine. The daily dose in the MYOMEX trial started at 200 mg for 3 days, at which point all patients had a dose titration up to 400 mg for a further 3 days and then a final titration to 600 mg for 12 days, at which point efficacy was assessed. The summary of product characteristics for Namuscla states that the dosing schedule is based on clinical response and can be increased after at least 1 week of treatment in 167 mg (200 mg imported mexiletine dose equivalent) increments to a maximum dose of 500 mg (600 mg equivalent). The clinical experts stated that the rapid forced dose titration to 600 mg in MYOMEX does not represent current clinical management and is not in line with the summary of product characteristics. Currently, some people have dose titration in smaller off-label 100 mg dose increments at a more cautious rate of titration to avoid gastric side effects of mexiletine. Some people who are experienced with mexiletine use could have a faster rate of titration, but the clinical experts considered that this would not be as fast as in MYOMEX. The committee considered that because of the short duration of the MYOMEX trial, some adverse events might not have been reported. In clinical practice, such adverse events could take much longer than the MYOMEX trial duration to emerge. The

	clinical experts stated that most patients currently have between 300 mg and 400 mg of imported mexiletine but patients with more severe symptoms, or patients with specific subgroups of myotonia that need greater doses, can have 600 mg doses or greater. The company considered the average daily dose of 417 mg in the Suetterlin et al. retrospective review to be the most accurate dose for modelling, and therefore included 15 capsules a week (equivalent to a daily dose of 429 mg) in its base case. The committee noted the difference between this dose and the 600 mg dose that was used at the point of assessment of efficacy in MYOMEX. It considered that it is not usually appropriate to separate the costs and benefits of treatments. The company stated that people in MYOMEX had the opportunity to immediately continue treatment with mexiletine at a dosage adapted to their clinical response and tolerance to the drug, after the initial titration to 600 mg. The company explained that the average dose used in clinical practice at the largest treating centre in the UK was 300 mg to 400 mg, with 600 mg not usually needed to reach maximum quality-of-life improvements. The company stated that the experts it consulted with had estimated that 400 mg was the average dose in clinical practice. The committee decided it was appropriate to consider the costs of the 429 mg dose (informed by Suetterlin et al. and clinical expert opinion on current practice). However, it also considered a scenario with the costs of the 600 mg dose (as was seen in MYOMEX), because it was mindful that efficacy estimates in the trial were taken once treatment had been titrated up to the 600 mg daily dose, so there would be uncertainty around the clinical-effectiveness results. The committee concluded that the dose and dosing schedule in MYOMEX does not reflect how mexiletine is currently used or would be used in clinical practice, so the cost of mexiletine is uncertain."
Top 9 (text471)	"3.11 The economic model was developed using a Markov structure, comprising 9 health states defined by the days of parenteral support per week (from 7 days to parental support independence or to death). The company included a treatment stopping rule so that modelled teduglutide use would reflect its use in clinical practice as closely as possible. The summary of product characteristics recommends that treatment should be stopped if there is no overall improvement in the condition. It recommends that adults should have an evaluation after 6 months, with treatment continuation being reconsidered if there is no treatment benefit by 12 months. The model reflected this by assuming that those who had not had a reduction of at least 1 day of parenteral support per week at 12 months, compared with baseline, stop teduglutide. Once treatment is stopped, they immediately reverted to their baseline parenteral support state before teduglutide. Teduglutide is modelled to affect both cost and quality-adjusted life years (QALYs): Costs: Drug treatment (teduglutide) costs are increased. Costs associated with parenteral support, concomitant drugs, and complications linked to parenteral support are reduced. Incidence of adverse events are changed compared with standard care., QALYs: The number of days that people need parenteral support per week is reduced. This is modelled to improve the health-related quality of life of people with SBS and their carers. The incidence of complications associated with parenteral support are reduced. There are carer benefits. To calculate transition probabilities for teduglutide, the company pooled clinical data from the teduglutide arms of STEPS and STEPS-2 and data from the PSP when estimating the reductions in parenteral support for the teduglutide group. It explained that it took this approach rather than using the relative treatment effect from the trial because the weaning algorithm in STEPS and STEPS-2 underestimates parenteral support reductions for teduglutide (see section 3.8). The company supported this claim by doing an analysis comparing the percentage of people stopping parenteral support entirely while taking teduglutide between STEPS, PSP, and a combination of other real-world studies. The company also assumed that there is no change in parenteral support in the standard care arm and applied the STEPS baseline parenteral support requirement over the time horizon in the standard care arm of the model. The reasoning for this was that people need to have a stable parenteral support requirement before teduglutide, and reductions in parenteral support would not be expected in clinical practice without teduglutide (see section 3.3). The ERG confirmed that the model structure is appropriate. It advised that the company's explanation for underestimation of teduglutide effectiveness in the STEPS and STEPS-2 trials was plausible, but that any comparison of effects between observational studies and randomised controlled trials should be interpreted with caution. The committee expressed some concern around the company's methodology for estimating transition probabilities. This was specifically related to breaking randomisation when pooling the real-world and teduglutide arm trial data while disregarding the relative treatment effect and placebo data from STEPS. The ERG stated that it had done a scenario analysis exploring the relative treatment effect of teduglutide from the STEPS data alone. This had a substantial upwards impact on the incremental cost-effectiveness ratio (ICER). But because it received clinical expert feedback that people having standard care would not be expected to reduce their parenteral support needs, the ERG considered this scenario to be conservative and did not incorporate it into its base case. At its first meeting, the committee concluded that the company's approach to modelling health-state transitions in both arms was a source of uncertainty and requested further scenario analyses. In response to these concerns, the company provided 2 scenarios: Using STEPS placebo arm data to calculate the first 6 months of transitions within the standard of care arm of the adult base case (only 6 months was considered by the company because it did not consider the placebo effect in STEPS to be sustainable long term). Using only data from STEPS or STEPS-2 in the teduglutide arm of the adult base case, rather than pooling data from STEPS, STEPS-2 and PSP. Both these scenarios had a modest upwards impact on the ICER. The ERG combined the 2 scenarios but stated that this was pessimistic and probably underestimated the benefit of teduglutide. The committee agreed that these scenarios resolved some uncertainty around the

	calculation of transition probabilities in the model. It concluded that the transition probabilities were a source of uncertainty but were appropriate for decision making."
Top 10 (text266)	"3.6 The company used data from OTUS to update its stage 1 model at technical engagement. OTUS is a retrospective real-world evidence analysis of CMV infection that is refractory or resistant to treatment, with a longer follow up than SOLSTICE. The company used the OTUS data to populate the model beyond the 20-week duration of SOLSTICE. This included modelling recurrences for the first 20 weeks based on SOLSTICE data, then using OTUS data to model outcomes for the remaining stage 1 time horizon. The ERG considered OTUS to be more generalisable to clinical practice than SOLSTICE, but had concerns with the way the company used the OTUS data, which assumed that the populations and outcomes in OTUS and SOLSTICE were interchangeable. The ERG highlighted that the ratio of SOT to HSCT procedures, percentage of clearance, and time since transplant differed between the 2 sources. The ERG preferred to use OTUS to model the probability of clearance and recurrence for IAT in the stage 1 Markov model, with the outcomes for maribavir estimated by applying a relative treatment effect taken from SOLSTICE. OTUS could also be used to inform risk of mortality, time since transplant and event rates of complications such as graft failure and graft-versus-host disease. In a scenario analysis done by the company using the OTUS data, clearance rates were adjusted for 8-week mortality. The ERG was unclear about why this had been done, and preferred to use data that had not been adjusted for mortality at 8 weeks. The committee preferred the ERG's approach. At the first meeting, it agreed that using OTUS data as far as possible, with the relative treatment effect of maribavir from SOLSTICE, would be more robust for modelling outcomes in the stage 1 Markov model, and that data from OTUS should not be adjusted for mortality at 8 weeks. In response to consultation, the company incorporated OTUS data in its revised analyses, with the relative treatment effect of maribavir from SOLSTICE. The company noted the uncertainties of incorporating 2 data sources in the model, but maintained that SOLSTICE was the most reliable data source to estimate the treatment effect of maribavir compared with standard care. The ERG commented that the company had not provided the underlying data for clearance events for the SOT population, and queried the company's estimate of probability of clearance for the HSCT population. Ahead of the second committee meeting, the company submitted additional data from OTUS. The ERG was satisfied with the company's update and noted that it had a minimal effect on the incremental cost-effectiveness ratio (ICER). The committee concluded that the data used in the company's model was suitable for decision making."

Supplement 7 – univariable regression results

Table 7: Univariable binary logistic regression models with uncertainty paragraphs as dependent variable (Lasso model, stemmed DFM, base case threshold of 0.5, N=4958)

Covariate	Level	OR	95% CI*	Clustered SE	p-value*
Guidance					
	TA[a]	-	-	-	-
	HST	1.60	1.26, 2.03	0.092	<0.001
ATMP status					
	No[a]	-	-	-	-
	Yes	1.24	0.96, 1.60	0.100	0.160
Disease area					
	Oncology[a]	-	-	-	-
	Other	1.35	1.10, 1.66	0.080	<0.001
Age group					
	Adults[a]	-	-	-	-
	Children	1.42	1.05, 1.93	0.119	0.016
	Both	1.31	1.01, 1.69	0.100	0.037

[a] = reference level; AOR = adjusted odds ratio; ATMP = advanced therapy medicinal product; CI = confidence interval; DFM = document-feature matrix; HST = Highly specialized technology appraisal guidance; SE = standard error; TA = Technology appraisal guidance
* Bonferroni adjusted confidence intervals and p-values (No. of hypotheses = 5)

Supplement 8 – classification performance across thresholds

Figure 3: Accuracy performance per threshold value for all models (stemmed DFM, N=4958)

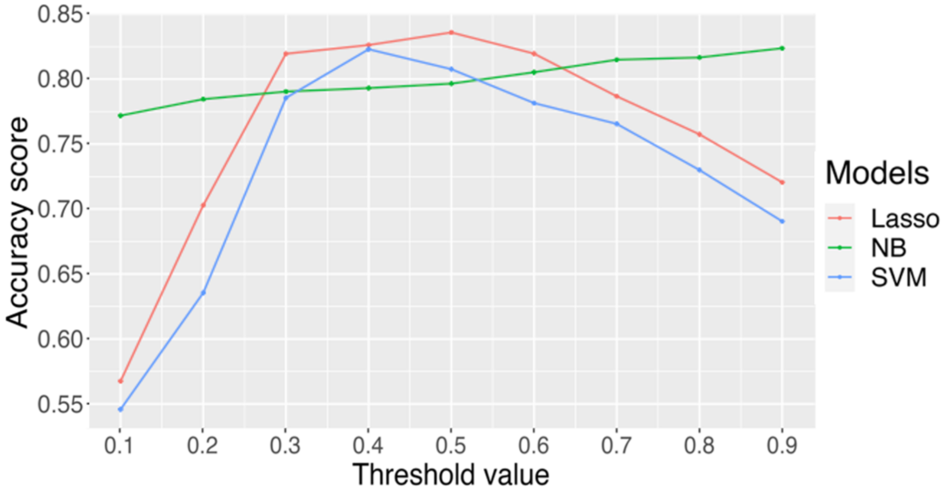

NB = Naïve Bayes, SVM = Support Vector Machines

Figure 4: Sensitivity performance per threshold value for all models (stemmed DFM, N=4958)

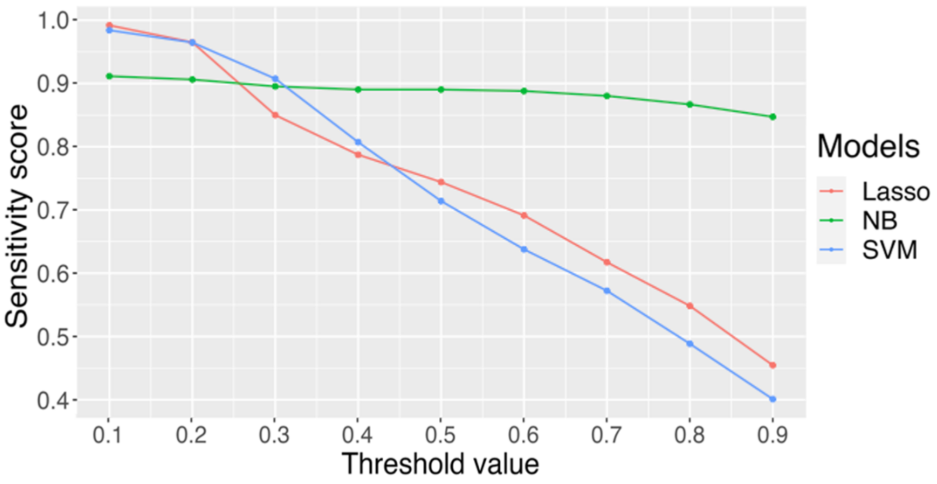

NB = Naïve Bayes, SVM = Support Vector Machines

Figure 5: Specificity performance per threshold value for all models (stemmed DFM, N=4958)

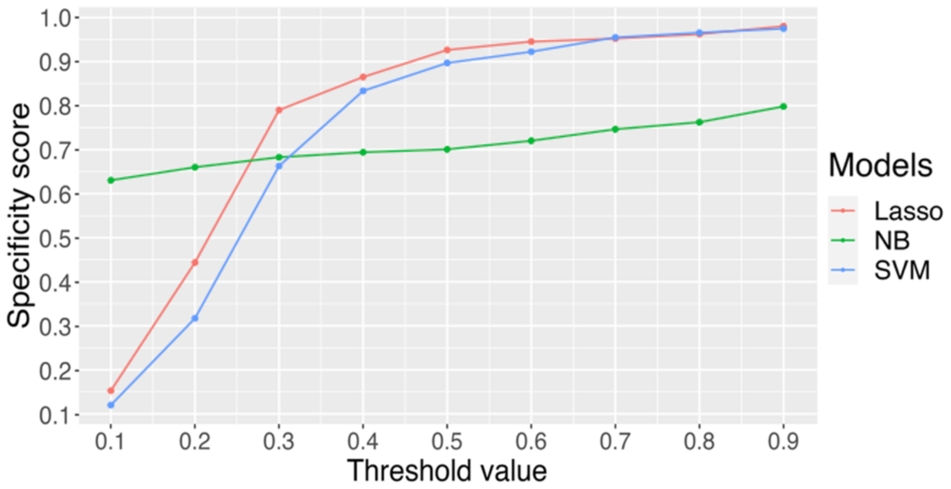

NB = Naïve Bayes, SVM = Support Vector Machines

Supplement 9 – multivariable regression results across thresholds

Figure 6: Adjusted Odds Ratios (AORs) of multivariable logistic regression analyses with uncertainty paragraphs as dependent variable at different threshold values for the probability of classifying paragraphs as uncertainty paragraphs (Lasso model, stemmed DFM, N=4958)

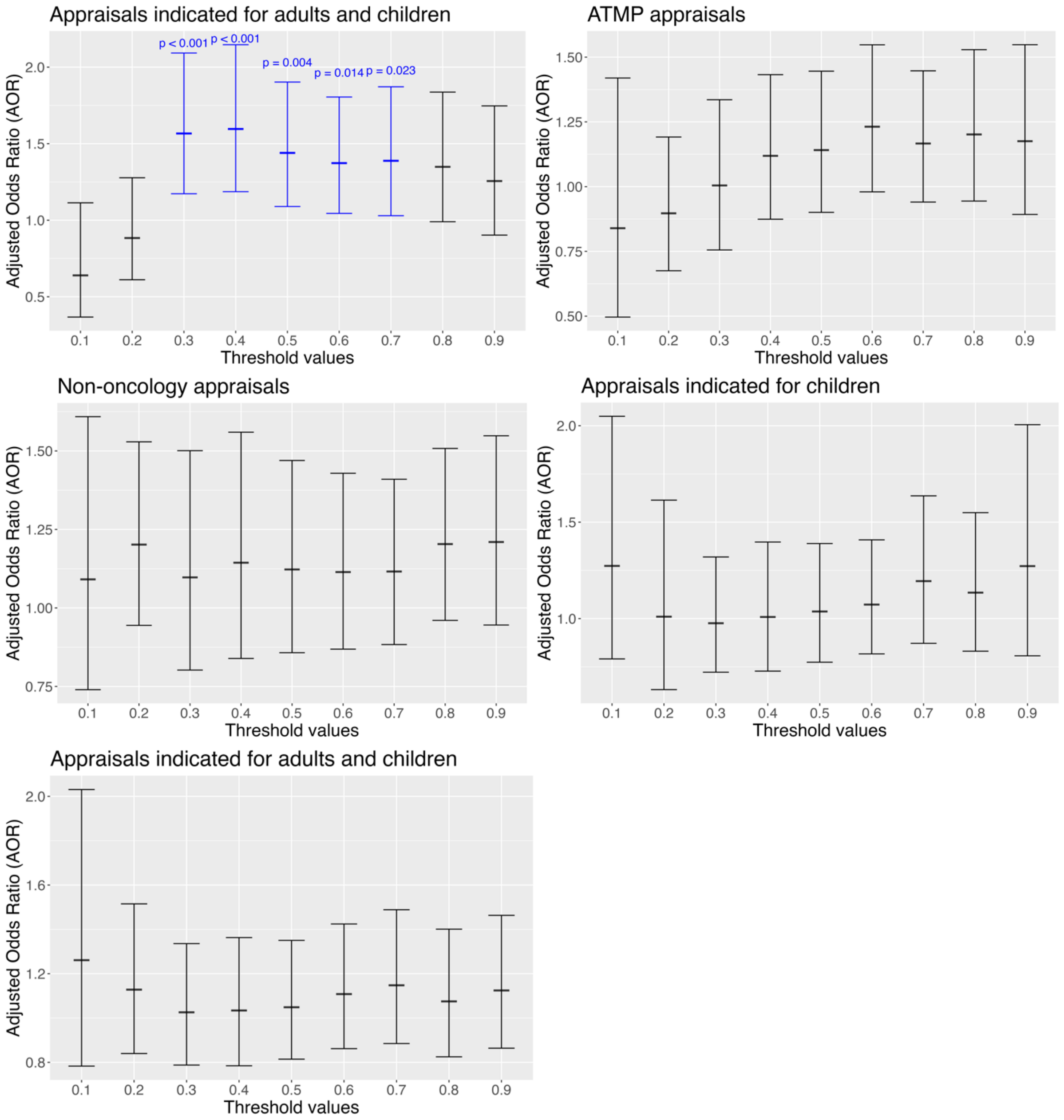

AOR = adjusted odds ratio; HST = Highly specialized technology appraisal guidance; p = p-value
Bonferroni adjusted confidence intervals and p-values (No. of hypotheses = 5)
AORs, confidence intervals and p-values < 0.05 are highlighted in blue

Supplement 10 – multivariable regression results across models

Table 8: Number of paragraphs classified as referencing uncertainty across different models (stemmed DFM, base case threshold of 0.5, N=4958)

Model	Number of paragraphs classified as referencing uncertainty (%)
Lasso	1952 (39.37)
Naïve Bayes	2872 (57.93)
SVM	2127 (42.90)

SVM = Support Vector Machines

Table 9: Multivariable logistic regression model with uncertainty paragraphs as dependent variable (SVM model, stemmed DFM, base case threshold of 0.5, N=4958)

Covariate	Level	AOR	95% CI*	Clustered SE	p-value*
Guidance					
	TA[a]	-	-	-	-
	HST	1.38	0.92, 2.09	0.160	0.215
ATMP status					
	No[a]	-	-	-	-
	Yes	1.03	0.72, 1.46	0.137	1.000
Disease area					
	Oncology[a]	-	-	-	-
	Other	1.17	0.82, 1.68	0.139	1.000
Age group					
	Adults[a]	-	-	-	-
	Children	1.50	0.93, 2.41	0.185	0.147
	Both	1.10	0.75, 1.61	0.147	1.000

[a] = reference level; AOR = adjusted odds ratio; ATMP = advanced therapy medicinal product; CI = confidence interval; DFM = document-feature matrix; HST = Highly specialized technology appraisal guidance; SE = standard error; TA = Technology appraisal guidance
* Bonferroni adjusted confidence intervals and p-values (No. of hypotheses = 5)
Model adjusted for guidance type, ATMP status, disease area, and age group

Table 10: Multivariable logistic regression model with uncertainty paragraphs as dependent variable (Naïve Bayes model, stemmed DFM, base case threshold, N=4958)

Covariate	Level	AOR	95% CI*	Clustered SE	p-value*
Guidance					
	TA[a]	-	-	-	-
	HST	0.82	0.58, 1.17	0.137	0.750
ATMP status					
	No[a]	-	-	-	-
	Yes	0.96	0.71, 1.30	0.116	1.000
Disease area					
	Oncology[a]	-	-	-	-
	Other	1.04	0.74, 1.46	0.131	1.000
Age group					
	Adults[a]	-	-	-	-
	Children	1.19	0.79, 1.78	0.157	1.000
	Both	1.19	0.89, 1.60	0.115	0.620

[a] = reference level; AOR = adjusted odds ratio; ATMP = advanced therapy medicinal product; CI = confidence interval; DFM = document-feature matrix; HST = Highly specialized technology appraisal guidance; SE = standard error; TA = Technology appraisal guidance
* Bonferroni adjusted confidence intervals and p-values (No. of hypotheses = 5)
Model adjusted for guidance type, ATMP status, disease area, and age group
